# Acoustic Performance of 3D Printed Nanocomposite Earmuff

**DOI:** 10.5539/gjhs.v8n1p180

**Published:** 2015-05-15

**Authors:** Saeid Ahmadi, Parvin Nassiri, Ismaeil Ghasemi, Mohammad R. Monazzam Ep

**Affiliations:** 1 School of Public Health, Tehran University of Medical Science, Tehran, Iran; 2 Plastic Department, Process Research Center, Iran Polymer and Petrochemical Institute, Tehran, Iran

**Keywords:** ABS/nanoclay composite, single and double cup earmuffs, insertion loss, three dimensional printer

## Abstract

**Introduction:**

Hearing protection devices are one of the primary noise reduction tools in developing countries. This study is intended to produce and apply acrylonitrile butadiene styrene (ABS)/clay nanocomposites to fabricate a laboratory single cup earmuffs and then compare it with double cup and single cup pure ABS earmuffs in terms of noise attenuation performance and comfort. In addition, the noise attenuation performance of single cup pure ABS earmuffs is compared with double cup pure ABS earmuffs.

**Methods:**

ABS/nanoclay filament was fabricated using a twin screw extruder. A three dimensional (3D) printing machine and a 3D model of earcup, designed by solid work software, were applied to print single and double cup earmuffs using ABS/nanoclay composite and pure ABS filaments. Finally, using an acoustic test fixture, objective noise attenuation test was performed on three different types of earmuffs, including with and without nano material and a secondary cup. Moreover, earmuffs weight was measured as a comfort component.

**Results:**

Insertion loss and calculated noise reduction rating (NRR) of single cup ABS/nanoclay earmuffs (NRR=19.4 dB) and double cup pure ABS earmuffs (NRR=18.93 dB) were improved in comparison with single cup pure ABS earmuffs (NRR=15.7 dB). Additionally, both single cup earmuffs were significantly lighter than double cup earmuffs. Although single cup nano and double cup earmuffs had nearly the same attenuation performance, single cup nano earmuffs were 74 gr lighter than double cup earmuffs, so with reference to comfort, single cup nano earmuffs will probably be more acceptable.

**Conclusions:**

From this survey it might be concluded that, even though single cup ABS/nanoclay earmuffs was lighter than double cup pure ABS earmuffs, it had approximately more attenuation performance in comparison with double cup pure ABS earmuffs. Consequently, users are probably more prone to wear light- weight single cup ABS/nanoclay earmuffs as a result of improved comfort. In short, ABS/nanoclay composite can be considered a good choice in products with the necessity of high acoustic performance and low weight.

## 1. Introduction

Utilizing hearing protection devices (HPDs) are the most cost-effective method for personal exposure control; moreover, wearing hearing protectors are of primary importance in many areas. On the other hand, it should be used where there is a need to provide additional protection beyond what has been achieved through technical and organizational means (Dietz et al., 2005; Buck, 2009). Furthermore, engineering noise controls and programs are not considered in most of developing countries as a result of old equipment, economical and cultural issues, thus HPDs have to be designed so that that they satisfy employees (Neitzel et al., 2005).

Comfort and noise attenuation are the leading characteristics of hearing protectors. However hearing protection comfort, education, and motivation of workers persuade them to wear HPDs for longer times, users feel uncomfortable after wearing heavy and uncomfortable hearing protection devices for a short period of time. If workers find earmuffs uncomfortable, they will be less eager to wear them ceaselessly, thus comfortable HPDs can influence users’ tendency to wear them for a longer time, so the main target, which is mitigation of employee’s exposure, will be achieved eventually. As a result, comfort factor is more important than noise attenuation. In addition, protectors with lower labeled attenuation but with higher comfort are considered to be more efficient than protectors with higher labeled attenuation but lower comfort (Hsu et al., 2004; Nélisse et al., 2012).

Soundproofing and weight are the fundamental components of comfort for earmuffs and on top of that comfort of hearing protectors are directly related to the weight (Gerges, 2012). If the weight of an HPD increases, the force of gravity which pushes the device against the head can be strongly increased in a way that reduces comfort. Moreover, the movement of the head can dislocate a heavy hearing protector due to the inertia effect. A good hearing protector encompasses both high noise attenuation and high comfort level (Davis, 2008; Gerges, 2012). Regarding attenuation, HPDs are commonly described by ratings such as the noise reduction rating (NRR). These ratings are remarkably used to estimate the workers effective exposure (Gerges et al., 1995; Nélisse et al., 2012).

For extreme noise environments, high attenuations close to 45-50 dB are highly preferred. Generally, to achieve higher attenuation both earplugs and earmuffs need to be used in combination, forming so called double hearing protection (DHP). DHP systems are recommended or sometimes mandated by authorities (e.g., National Institute for Occupational Safety and Health -NIOSH) in situations where the noise level is higher than a pre-determined limit (for example, 104 dBA). Unfortunately, due to inconvenience and complexity associated with the donning procedure, DHP systems are not always accepted by wearers (Behar et al., 1999; Du et al., 2009). Therefore, double cup hearing protectors are recommended by manufacturers as an alternative for double hearing protection. Although double cup hearing protectors have a satisfactory level of noise attenuation, they are heavy. An earmuffs weight less than 245 g is acceptable by most users while double cup hearing protectors weight is more than 245 g which cannot be accepted in term of weight; such a weight is too high to feel comfort while wearing them. The weight of double cup hearing earmuffs are approximately more than 300 gr. Weight of muffs accounts for 68-77% of the total weight, which should be the major target if a light weight design is desired. Therefore, it may be desirable to maintain or improve the attenuation of earmuffs by means of new material and design, and simultaneously improve its comfort so that it can be used in higher noise environments (Hsu et al., 2004).

Polymer-clay nanocomposites have shown remarkable sound insulation characteristics together with light quality and high strength at low filler loading. Therefore, wherever good noise insulation tools, such as hearing protection devices, are needed commercial applications of these materials seem to be promising. In addition, this study is a response to one of the research needs of NIOSH, i.e. “application of nanomaterials and nanotechnology in the field of occupational safety and health” (Lee et al., 2008; Aliabadi et al., 2014a; Yan & Kim et al., 2014).

This study is intended to produce and apply ABS/clay nanocomposites to fabricate a laboratory single cup earmuff prototype. Then these single cup nano earmuffs will be compared with double cup and single cup pure ABS hearing protectors in terms of noise attenuation performance and comfort. In addition, the noise attenuation performance of single cup pure ABS is compared with double cup pure ABS.

## 2. Methods

### 2.1 Materials

The ABS polymer with the trade mark of SD0150 was applied as a matrix in this study. It was supplied by Tabriz Petrochemical Co, Iran. Its density and melt flow index were 1.05 g/cm^3^ and 1.8 g/10 min, respectively. An inorganic particle was applied as nanofiller. It was obtained from Southern Clay Products Co. (Texas, USA) with the trade name of Cloisite 30B. The size and density of the nano particle were ≤ 13 μm and 1.98 g/cm^3^ respectively.

### 2.2 Sample Preparations

ABS/clay nanocomposites were prepared by melt blending using a twin screw extruder (Brabender, Germany). The nanocomposites were prepared at a temperature profile of 170–200°C and at a screw speed of 80 rpm. The continuous filaments from the extruder were dried before 3D printing. In this study, specimens were prepared at 4, wt. % filler concentrations.

**Figure 1:**
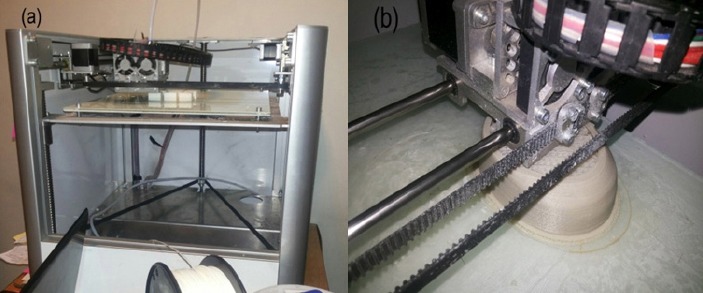
Three dimensional (3D) printing machine

A 3D printing machine (Model: Creater, Leapfrog, Netherlands) was used to prepare test specimens. These types of 3D printers run on fused deposition modeling (FDM) technology. The process includes pre-processing, production, and post-processing. Firstly, in the pre-processing step, build-preparation software sliceed and positioned 3D file of ear cups, that had been already designed by solid work software, and calculated a path to extrude filament. Secondly, in the production step, the 3D printer heated the filament and deposited it along the extrusion path. Thirdly, in the post processing step, we broke support material or dissolved it in a solvent (acetone). Finally, specimens surface were attractively finished by polishing and painting in order to smooth down the surfaces. Figure 1 illustrates a view of 3D printing machine (a) and during printing earcup (b).

### 2.3 Insertion Loss Measurements

Insertion loss (IL), as an acoustical measure, is commonly related to the attenuation of noise in hearing protection devices. IL is the algebraic difference, in decibel, between the one-third octave band sound pressure levels (SPLs) measured at a reference point in a specified sound field under specified conditions before and after noise treatment. An example is sampling sound pressure inside the ear canal, with (P) and without (P’) HPD in place. It is described in Equation 1 (de Almeida-Agurto et al., 2011).


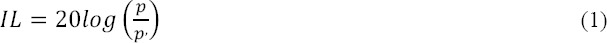


Hearing protector attenuation performance can be measured using acoustical test fixture (ATF) that is an objective method (Berger, 2005). The ATF used in this study (type 45CA, G.R.A.C, Denmark) was a binaural implementation of a mannequin with one instrumented ear (left ear). The mannequin’s features included a resilient base, circumaural areas, test fixture head, and acoustic plug. The pink noise signal used for the tests was generated by an on-board Norsonic noise generator type Nor140 and fed into the measurement room via Microlab speakers type Solo 6C. The resulting signals were detected by a G.R.A.C 1” pressure microphone - type 40EN and analyzed by a Norsonic real-time frequency analyzer type Nor140 equipped with 1/3 octave analysis from 31.5 Hz to 8 kHz.

**Figure 2:**
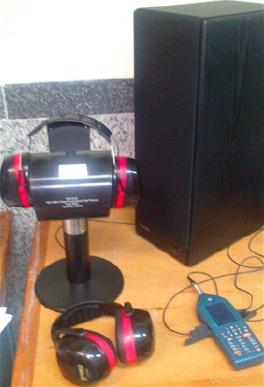
An illustration of acoustic test fixture (ATF) measurement system

The experimental study was performed according to the recommendations of the American National Standards Institute (ANSI S12.42-1995) and the International Organization for Standardization (ISO 4869-3 -2002). The attenuation tests of HPDs were conducted on the three groups of earmuffs. For each group, a series of three measurements were performed. For each signal frequency, the difference in sound pressure levels was considered as the insertion loss of the device. The arrangement used during the test is shown in Figure 2 (ISO, 2007; ANSI, 2010).

Finally, assumed protection value, APV_fx_ and NRR were calculated by the mean IL and standard deviations of earmuffs. In this study, the NRR was calculated using the IL and APV values. IL is a quantity that is directly related to the effectiveness of an HPD and is analogous to the hearing threshold shift (Behar et al., 1999).

### 2.4 Weighting Character

Soundproofing and weight are the fundamental components of comfort for earmuffs. Soundproofing was assessed by measuring insertion loss of various earmuffs in previous section. Weighting character, as a component of comfort index, was assessed by measuring the weight of fabricated earmuffs. All single and double cup earmuffs with nano and pure ABS had the same shape, thickness, and volume in cups. Despite these similar futures, we expected to have different mass as if nano clay and pure ABS had different densities. So, in order to compare them in terms of comfort, the weight of all specimens were measured by an AND (Japan) laboratory scale type GR200.

## 3. Results

### 3.1 Three Dimensional Model and Prototypes

**Figure 3:**
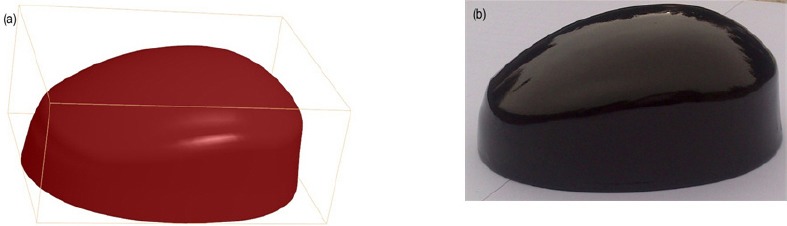
Three dimensional solid model of ear cup in solid work (a) and a prototype of printed ear cup (b)

ABS/nanoclay and pure ABS filaments were fed into the 3D printer to produce a prototype of ear cup with and without nano filler, respectively. The ear cup geometry, that was designed by solid work software, was illustrated in Figure 3(a). An ear cup printed with nanocomposite filaments is shown in Figure 3(b). All ear cups were painted with the same black color and there was no difference between printed earcups on the base of appearance.

### 3.2 Noise Attenuation

A summary of the attenuation results for single cup pure ABS earmuffs, single cup ABS/nanoclay earmuffs and double cup pure ABS earmuffs for each frequency is presented in Table 1. The last column corresponds to NRR which was calculated using the IL values.

**Table 1:** Summary of insertion loss (IL), standard deviations and the calculation of APV_f_ (dB)

		1.25KHz	2.5KHz	5KHz	1KHz	2KHz	4KHz	8KHz	NRR(dB)
Single cup- Pure ABS	m_f_ (IL)	5.7	8.1	11.9	21	22.2	38.2	43	15.7
	s_f_	0.2	0.3	0.1	0.8	2.4	3.5	4.6	
	αs_f_(α=0.84)	0.16	0.25	0.08	0.67	2.01	2.94	3.86	
	APV_f80_	5.53	7.84	11.81	20.32	20.18	35.26	39.13	
Single cup - ABS/Nanoclay	m_f_ (IL)	6.1	11.4	15.9	25.7	29.4	46	52	19.4
	s_f_	0.2	0.5	0.4	1.2	1.2	3.6	3.9	
	αs_f_(α=0.84)	0.16	0.42	0.33	1.00	1.00	3.02	3.27	
	APV_f80_	5.93	10.98	15.56	24.69	28.39	42.97	47.72	
Double cup-pure ABS	m_f_ (IL)	5.8	11.3	15.2	24.3	29.7	45.5	49	18.93
	s_f_	0.1	0.3	0.6	0.9	1.5	4.1	3.8	
	αs_f_(α=0.84)	0.08	0.25	0.50	0.75	1.26	3.44	3.19	
	APV_f80_	5.71	11.04	14.69	23.54	28.44	42.05	45.80	

**Figure 4:**
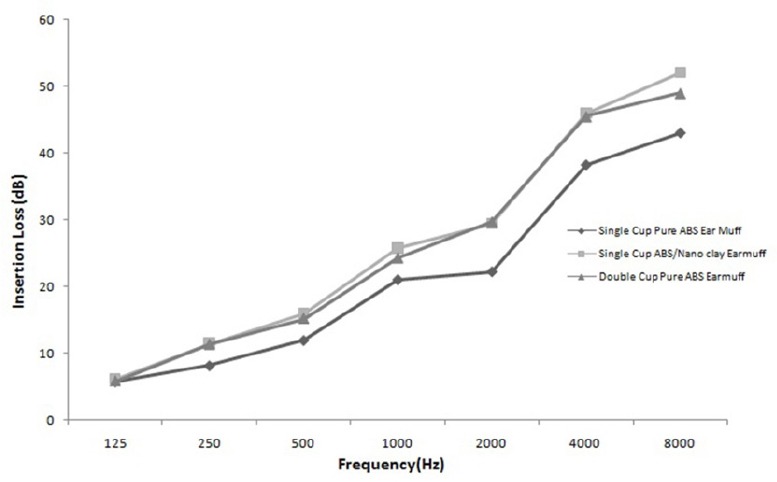
Results of insertion loss measurement for single and double cup earmuff

Figure 4 shows the average attenuation of three measurements performed on earmuffs with and without nanoclay and secondary cup. It can be seen that the largest increase in ILs were found at high frequencies in all earmuffs and there is almost no change in 1.25 KHz.

Attenuation for the single cup pure ABS earmuffs increased from 5.7 dB at 1.25 KHz to 21 dB at 1 KHz then remained fairly stable at approximately 22 dB from 1-2 KHz and finally increased sharply from 22.2 dB at 2 KHz to 43 dB at 8 KHz. The single cup pure ABS is an earmuffs with a linear frequency response and a relatively low (NRR=15.7 dB) attenuation. On the other hand, double cup pure ABS earmuffs attenuation increased monotonically from 5.8 dB at 1.25 KHZ to 49 dB at 8 KHz. On average, in all frequency bands except for 1.25 KHz, double cup ear muffs had about 3-8 dB higher IL than single cup pure ABS earmuffs. Also, as we expected, double cup earmuffs had more attenuation (NRR=18.93 dB) than single cup pure ABS earmuffs.

Single cup nano earmuffs had attenuation values in the range of 6.1 to 52 dB with highest values at 1 KHz (25.7 dB) and 8 KHz (52 dB). On average, the IL of single cup nano earmuffs was about 3.5–9 dB higher than single cup pure ABS earmuffs. Here, again there was no significant change at 1.25 KHz and a very bigger difference at 1 KHz and 8 KHz. The increase of ILs was relatively higher than previous case in some frequencies such as 1 KHZ and 8 KHz. The difference was more significant at frequencies higher than 1 KHz. Finally, single cup ABS nanoclay earmuffs had approximately the highest attenuation (NRR=19.4 dB) among earmuffs.

### 3.3 Weighting and Comfort

Weighting character is considered as a main component of earmuffs comfort. Table 2 shows the average of weight measurement performed on earmuffs. As it can be seen the double cup pure ABS earmuffs had the highest weight (310 gr) and as well as that single cup nano and pure earmuffs were the lightest (236 gr).

**Table 2:** Results of testing earmuffs attenuation and weight

Earmuff Specimens	Weight (Mean ± SD)	NRR (dB)
Single cup - nano	236 ± 5 gr	19.4
Single cup - pure	236 ± 4 gr	15.7
Double cup - pure	310 ± 3 gr	18.93

## 4. Discussion

### 4.1 Three Dimensional Model and Prototypes

*Generally, 3D printer which used in this study, is fed with the special material that is recommended by manufacturer, such as acrylonitrile butadiene styrene* (*ABS*), polylactic acid (PLA) *and polyvinyl alcohol (PVA). Even though, ABS/nanoclay filaments were fed into the printer, 3D printed earcups had feasible structure and form*. In order to have a rigid ear cup the 3D printer was set on the most accurate operation in a way that printer injected a layer of melted nanocomposites on another layer with a negligible gap between layers. Surprisingly, It was shown that ear cups with ABS/nanoclay had a more smooth surface in comparison with ear cups with pure ABS.

In spite of the fact that earcup specimens were successesfully printed by ABS nanocomposite, challenges in the application of nanocomposites with 3D printers are various: hardly ever nanocomposites have been fed into the 3D priter; there is a relative lack of knowledge on the interaction of nanofillers with 3D printing material; and operational parameters and fabrication techniques for different nanocomposites have not been set as a standard yet. Our results were in accordance with the past studies about incorporating nanocomposites into 3D printer (Campbell et al., 2013).

Few companies rely on 3D printing technology to create attractive, accurate prototypes that function realistically and give designers a chance to see the final product before production. Peltor company (USA) is a leading manufacturer of hearing protectors that benefit from 3D printers during product development. Peltor utilizes printers with polymer jetting technology that is more advanced and expensive than FDM technology (Fischer, 2013).

### 4.2 Noise Attenuation

In order to evaluate the effect of nano material on insertion loss, the average attenuation of single cup earmuffs with pure ABS was comparable to single cup earmuffs with nanoclay. In addition, in order to evaluate the effect of secondary cup on insertion loss, the average attenuation of single cup pure ABS earmuffs was comparable to double cup pure ABS earmuffs. Moreover, in order to characterize earmuffs, the average attenuation of single cup ABS/nanoclay earmuffs was comparable to double cup pure ABS earmuffs. Single cup ABS/nanoclay earmuffs can be a proper alternative to double cup pure ABS earmuffs because it provides higher comfort and attenuation performance. Also it is worth mentioning that all earmuffs had the same shape, inner volume, and thickness. Therefore, nano filler, secondary cup, and weight were specific variables that could influence attenuation ratings of earmuffs in this study.

In comparison with single cup pure ABS, single cup nano earmuffs are more protective at frequencies higher than 1 KHz. So it can be seen that nano filler are more efficient in higher frequencies. Sound insulation efficiency of nanoclay reinforced polymer composites has confirmed that polymeric nanocomposites had more efficient soundproofing performance in high frequencies rather than low frequencies (Yan, Kim et al., 2014). Low attenuation values were obtained for single cup nano earmuffs less than 1 KHz while it almost doubled after 1 KHz, suggesting the frequencies that weaknesses of earmuffs can be appeared with reference to the previous studies (Nélisse et al., 2012).

A mechanical rely is considered as a noise source in automobiles and electrical devices. Rely cases were made by ABS/carbon nano tube (CNT) and pure ABS in order to measure the soundproofing characteristic. The average insertion loss of ABS/CNT nanocomposite was higher than pure ABS by 3% (1.5 dB) (Lee et al., 2008). In our study, the average insertion loss of single cup nano earmuffs at frequencies 1-8 KHz was about 21-22% (4.7-9 dB) higher than single cup pure ABS earmuffs. Two dimensional structure of nanoclay are more soundproofing than one dimensional structure of CNT (Yan, Kim et al., 2011).

The reinforcement in noise insulation property of these single cup ABS/nanoclay earmuffs can be attributed to good dispersion and two dimensional structure of nanoclay which provided extended propagation route for the sound wave within the matrix in a way that many times of the reflection, scatter, refraction, and diffraction of the sound wave take place due to the the differences between the resin and filler density, leading to an increase in the sound wave propagation route and energy consumption. Moreover, nanoclay has a barrier effect against sound propagation route so, it takes longer time for passing earcups whereas sound energy will be dissipated due to passing this long and zigzag way. On the other hand, it is possible to damp the vibration of earcups due to damping effect of nanoclay (Anwar, 2005; Kim et al., 2013; Aliabadi et al., 2014b).

Hearing protectors weight is reported as the most important comfort component in some previous studies. Normally there is a direct relation between weight and attenuation in earmuffs (Arezes et al., 2008). ILs in single cup nano earmuffs were as large as double cup pure ABS, but there was a very small difference at 1 KHz and 8 KHZ frequencies. Moreover, the most important issue was that they had approximately the same hearing protection performance while they had different weights. The obvious weakness of double cup pure ABS earmuffs was that it was about 74 gr heavier than single cup ABS/nano earmuffs; therefore users won’t feel comfort due to wearing these quite heavy earmuffs. Our study showed that, although single cup ABS/nanoclay earmuffs had lighter weight in comparison with double cup earmuffs, there was no significant difference between their attenuation. The polypropylene (PP)/6.5 wt% nanoclay composite was nearly 2% heavier than pure PP that is negligible, while soundproofing efficiency of PP/6.5 wt% nanoclay was 52 % higher than pure PP (Kim et al., 2013; Yan, Lee et al., 2011). So it might be concluded that acoustical behavior of single cup ABS/nanocaly earmuffs are beyond the weight effect.

The attenuation performance of David Clark earmuffs was measured (NRR=22 dB) with a head acoustic mannequin. The averegae attenuation for David Clark earmuffs in frequencies less than 500 Hz was relatively suitable (20-25 dB) (Murphy, 2007). Our 3D printed earcups had poor performance (5.7-15.9 dB) in low frequencies (less than 500 Hz). Manufacturers utilize injection molding to produce commercial earmuffs, whereas we have have applied and tested 3D printed earcups that is a prototype of final products. Obviously, 3D printed earcups are not as rigid as injection molded earcups. So, injection molding machine will certainly improve rigidity of earcups with higher hearing protection performance.

### 4.3 Weighting and Comfort

According to the results (Table 2), single cup nano earmuffs were about 74 gr lighter than double cup pure ABS earmuffs wheares the attenuation performance of single cup nano (NRR=19.4 dB) and double cup pure ABS (NRR=18.93 dB)were almost the same. Users are more prone to wear lighter hearing protection devices since they are more comfortable. Previous studies have shown that 80 percent of wearers were more willing to wear hearing protectors that were lighter than 245 gr (Hsu, 2004).

Generally, uninstructed workers presume that hearing protectors with the highest noise reduction are the best and in most industrial environments hearing protector’s selection are usually based on just noise attenuation. Even though noise attenuation should be considered as a main factor, it should not be ignored that 90 % of occupational noise exposures are at a level less than 95 dB (A). As a result, hearing protectors with a lower weight and more comfort will be worn consistently and effectively (Byrne et al., 2011).

The effective performance of HPDs depends on how well and how long it is worn, so earmuffs’ weight, which is one of the leading factors influencing the comfort, may lead to misuse or intermittent use of HPDs. Also, protectors with lower labeled attenuation but with higher acceptiblity tend to be more efficient. As a result, single cup nano earmuffs will probably be more efficient in comparison with double cup pure earmuffs (Nélisse et al., 2012).

Moreover, it should be mentioned that removing the secondary cup provides bigger interior space, which improves heat-sinking efficiency of earmuffs and reduces inner temperature, so comfort will be increased indirectly (Hsu et al., 2004). In addition to improvement in comfort, previous studies have confirmed that bigger air volume inside earcups can increase the performance of single nano earcups (Murphy et al., 2007).

## 5. Conclusion

The present study was an attempt to examine noise attenuation performance and comfort of single and double cup earmuffs produced with ABS/nanoclay composite and pure ABS by using a 3D printer. An acoustic test fixture was applied to evaluate noise attenuation performance of various 3D printed earmuffs with and without nano material and a secondary earcups. Weight of 3D printed earmuffs was measured as a fundamental index of comfort. The result presented in the paper show that single cup ABS/nanoclay (NRR=19.4 dB) and double cup pure ABS (NRR=18.93 dB) earmuffs had better attenuation performance in comparison with single cup pure ABS (NRR=15.7 dB). Although there was no significant difference between attenuation performance of single nano and double cup pure ABS earmuffs, single cup nano was lighter (about 74 gr), consequently they are probably more eligible to be worn by users. The improvement in noise attenuation performance of single cup ABS/nanoclay earmuffs might be attributed to the blockage of sound propagation by two dimensional structure and good dispersion of nanoclay within the matrix. So single cup ABS/nanoclay earmuffs without a secondary cup will probably increase comfort and reduce production costs; moreover, its attenuation performance is similar to that of double cup pure ABS earmuffs.
